# Binding behavior of receptor binding domain of the SARS-CoV-2 virus and ivermectin

**DOI:** 10.1038/s41598-024-53086-0

**Published:** 2024-02-02

**Authors:** Kasidy R. Gossen, Meiyi Zhang, Zivko L. Nikolov, Sandun D. Fernando, Maria D. King

**Affiliations:** Department of Biological and Agricultural Engineering, Texas A&M University, 2117 TAMU, College Station, TX 77843 USA

**Keywords:** SARS-CoV-2, Kinetics

## Abstract

The COVID-19 pandemic, caused by severe acute respiratory syndrome coronavirus 2 (SARS-CoV-2), sparked an international debate on effective ways to prevent and treat the virus. Specifically, there were many varying opinions on the use of ivermectin (IVM) throughout the world, with minimal research to support either side. IVM is an FDA-approved antiparasitic drug that was discovered in the 1970s and was found to show antiviral activity. The objective of this study is to examine the binding behavior and rates of association and dissociation between SARS-CoV-2 receptor binding domain (RBD), IVM, and their combination using aminopropylsilane (APS) biosensors as surrogates for the hydrophobic interaction between the viral protein and human angiotensin-converting enzyme 2 (ACE2) receptors to determine the potential of IVM as a repurposed drug for SARS-CoV-2 prevention and treatment. The IVM, RBD, and combination binding kinetics were analyzed using biolayer interferometry (BLI) and validated with multiple in silico techniques including protein–ligand docking, molecular dynamics simulation, molecular mechanics-generalized Born surface area (MM-GBSA), and principal component analysis (PCA). Our results suggest that with increasing IVM concentrations the association rate with the hydrophobic biosensor increases with a simultaneous decrease in dissociation. Significant kinetic changes to RBD, when combined with IVM, were found only at a concentration a thousand times the approved dosage with minimal changes found over a 35-min time period. Our study suggests that IVM is not an effective preventative or treatment method at the currently approved dosage.

## Introduction

As of June 2023, there have been just over 767 million globally confirmed coronavirus disease 2019 (COVID-19) cases, and nearly 7 million deaths^1^. These numbers have continued to grow since the initial outbreak of pneumonia with unknown causes, which were later identified as COVID-19^2^. First reported in December 2019, in Wuhan, China the primary symptoms included chest pain, fatigue, dyspnea, and cough^3,4^. Due to all of the panic caused by the pandemic, numerous studies evaluating prevention, treatment, and vaccine studies relating to COVID-19 were performed within months of the outbreak and many investigations are still ongoing. There has been extensive testing on the various properties of severe acute respiratory syndrome coronavirus 2 (SARS-CoV-2), and Receptor Binding Domain (RBD) specifically. RBD is a crucial part of the spike glycoprotein (S protein) of the SARS-CoV-2 virus as it is responsible for receptor binding in the body as well as epithelial cells entry^5,6^. Tests including Enzyme-linked immunosorbent assay (ELISA), molecular docking and dynamics were performed to illustrate how the S protein and its RBD interact with the human Angiotensin-Converting Enzyme 2 (ACE2) receptor^7–9^. Since the initial outbreak, there have been numerous COVID-19 variants, many of which come from mutations of the RBD contained in the S protein^10^. In studies for RBD-based vaccines, the vaccine candidates have shown cross-neutralizing properties for different SARS-CoV-2 strains as well as inducing protective immunity^11^. However, with newer and stronger variants occurring, such as the Omicron strain, the effectiveness of vaccines targeting the original strain is relatively lower^12^. Therefore other methods for containing and reducing the effects of the virus are continuing to be explored.

Before COVID-19 vaccines became available, one of the early potential methods taken into consideration was ivermectin (IVM), which garnered support when a study published in April 2020 claimed that IVM inhibits the replication of SARS-CoV-2 in vitro^13^. It is believed that IVM is able to do this by binding to the importin α (IMPα) protein, which has the main purpose of transporting proteins into the nucleus, and inhibiting its function^14,15^. This, among other properties, led to IVM being considered for drug repurposing for the various COVID-19 variants as it would be a time and cost-effective method^16^. Potential roles of IVM against the virus include direct action, and action on host targets for viral replication and inflammation in various ways^17^. IVM is an antiparasitic drug, initially discovered in 1973, that won the Nobel Prize for Physiology or Medicine in 2015^18^. As an antiparasitic, specifically the class anthelmintic, it works by paralyzing and killing parasites with minimal effect on the host. It is approved for human use, in cases of infestations such as head lice and river blindness, as well as veterinary use for heartworms and other parasitic infestations but not an FDA-approved treatment for COVID-19. However, there were still people in the United States and other countries who continued to take IVM to prevent or treat SARS-CoV-2. For example, many Latin American and Caribbean countries were prescribing IVM as a preventative treatment for SARS-CoV-2, soon after the initial in-vitro results were published^19^. One study determined that 25 countries used IVM against COVID-19 to varying degrees, including 14 countries that have given nationwide approval for use^20^. The human-approved dosage is typically around 12 mg administered orally but testing has been performed on 200 μg/kg body weight to the relatively high dosage 600 μg/kg^21–26^. However, oral administration is not the only option, nasal spray, and nebulized options are being tested at varying dosages in humans, rats, and pigs to determine if IVM can be delivered directly to the infection site within the respiratory system^27–30^. Additionally, many in silico studies performed molecular docking and molecular dynamic (MD) simulations for the combination of IVM, SARS-CoV-2 RBD, and ACE2 receptors reported high docking score and suggested that IVM could potentially disrupt the RBD-ACE2 interactions^31,32^.

Our experiment was designed to supply the existing studies using the established dose for IVM treatment of 150–200 μg/kg and the average male weight in the United States of 90.6 kg, which is an equivalent approved dosage of 0.38 μM^33,34^. The objective of this study is to examine the binding behavior and rates of association and dissociation between SARS-CoV-2 RBD, IVM, and their combination to determine the potential of IVM as a repurposed drug for SARS-CoV-2 prevention and treatment. This was performed using Bio-layer interferometry (BLI), an optical technology that utilizes white light to determine various binding factors, such as binding, kinetics, and affinity in real-time^35^. Hydrophobic aminopropylsilane (APS) biosensors were selected to mimic the hydrophobic interaction between SARS-CoV-2 RBD and the human ACE2 receptors^36,37^. The samples were heated and the experiment was conducted at 37 °C to simulate the human body temperature. Additionally, we tested the interactions over a 0 to 35-min time frame in three intervals to determine if IVM would produce the desired kinetic results over a relatively short time period when combined with SARS-CoV-2 RBD. Our results showed that the desired kinetic changes to the RBD protein were only found at IVM concentrations of approximately 100 and 1,000 times the approved IVM dosage, suggesting that IVM is not an effective preventative or treatment method within the current dosage limit.

## Materials and methods

### Preparation of samples containing SARS‐CoV‐2 RBD

The materials for cell growth and purification of RBD were prepared following the same procedure by Zhang et al.^38^. RBD was expressed in HEK293 cells obtained from Dr. Jason McLellan, Dept. of Molecular Biosciences, The University of Texas, Austin and the Fc and His-tagged recombinant RBD was purified using the Ni-IMAC FF Sepharose column. Using the prepared RBD, which had an original concentration of 1.822 mg/ml and molecular weight of 58 kDa, and Phosphate-buffered saline (PBS), 1:2, 1:5, and 1:10 dilutions of RBD were prepared at 25 °C then stored at 4 °C in Eppendorf tubes. The 1:5 RBD dilution was then heated to 37 °C in a water bath and mixed with various IVM concentrations.

### Preparation of samples containing ivermectin

IVM in powder form (molecular weight of 0.8751 kDa) and PBS were used to create a 1:1 dilution of 380.5 μM concentration at 25 °C. Then a serial dilution was performed with PBS to create 1:10, 1:100, 1:1000, and 1:10,000 dilutions, also at 25 °C, and then stored at the same condition as the RBD samples.

### Determination of the basic kinetics for SARS‐CoV‐2 RBD

To determine and study the basic kinetics of the prepared samples of RBD of SARS-CoV-2 and IVM, BLI was performed on individual and mixed samples. The primary instrument used in this study was the Octet® R4 BLI Label-Free Detection system (Sartorius) and APS biosensors (Fortebio). The APS biosensors were hydrated with PBS in a 96-well black plate (Grenier) for at least 30 min before use. The samples were heated in a water bath at 37 °C for at least 20 min before plating 200 uL of each sample into a separate 96-well black plate. The combination samples were prepared by first adding the RBD into the plate and then the IVM before mixing with the pipette tip. The Octet® R4 system is capable of running four tests at a time, each following the same pathways and within a system set at 37 °C with the sample shake plate on. Each test contains a 60 s baseline step with PBS used as the buffer, a 300 s association step with the sample, and then 300 s for dissociation into PBS. It is important that at least one reference test is performed in each experiment by running the entire test with the PBS buffer. The Octet® CFR software was used to calculate and display all binding, association, and dissociation results. The mean and standard deviation of the kinetic constants were shown on the graphs.

### Statistical analysis

The one-way Analysis of Variance (ANOVA) was applied using JMP 17 to determine the statistical significance among three or more groups, *p*-values less than 0.05 were considered statistically significant. A comparison for all pairs using Tukey–Kramer HSD was also performed to determine the means significantly different from each other when applicable.

### Protein–ligand docking verification

The crystal structure of the spike protein receptor binding domain of SARS-CoV-2 in complex with ACE2 was obtained from the RCSB protein data bank (rcsb.org, PDB: 6VW1) at 2.68 Å resolution^39,40^. The RBD (Chain E) was extracted and prepared by the Protein Preparation Wizard in Schrödinger suite^41^. The polar hydrogen atoms were added to amino acid residues with valence errors and one missing side chain was fixed by Prime^42^. The IVM structure was obtained from PubChem^43^ and prepared by the LigPrep Wizard in Schrödinger suite^44^. Molecular docking was performed in Glide with the docking grid of size 10 × 10 × 10 Å set around the interaction site of RBD and ACE2 complex^45^. The protein–ligand docking poses and interactions were analyzed in Maestro^46^.

### Molecular dynamics

Molecular dynamics (MD) simulation was performed on the RBD and IVM conformation with the highest docking score using Desmond^47^. All simulations were performed in three replicates. An orthorhombic simulation box with minimized volume was generated to construct a solvated system using the System Builder in Desmond. Three commonly used explicit water models with three interaction sites—simple point charge (SPC), extended simple point charge (SPC/E), and transferable intermolecular potential 3P (TIP3P)—were initially built, and MD simulation with each model was performed for 10 ns to compare the effects of water models and determine which model to proceed for the longer simulation. The final MD simulation was built under the TIP3P model. Two chloride ions were used to neutralize the system and 0.1 M sodium chloride was added. Prior to the simulation, the system underwent the standard relaxation protocol to reach equilibration. Three protein–ligand complexes were tested—unbound RBD, and RBD with one and five IVM molecules. The full 100 ns MD simulation was performed with the OPLS3e force field in the isothermal-isobaric (NPT) ensemble at a temperature of 300 K and pressure of 1 atm for the recording interval of 100 ps which generated 1000 frames in total^48^. The protein α-carbon (Cα) root-mean-square deviation (RMSD) values were obtained with the Simulation Interactions Diagram Wizard in Desmond. The molecular mechanics-generalized Born surface area (MM-GBSA) method was used to calculate the binding free energy between the RBD protein and ivermectin over the 100 ns simulation. The MM-GBSA calculation was performed using the thermal_mmgbsa.py script in Schrödinger Prime which splits the trajectory file generated in Desmond into individual snapshots and computes the average binding energy of the frames^42^. The chosen calculation method used the VSGB 2.0 dissolution model and the OPLS3e force field^48,49^. The initial and final frame from the MD simulation of the RBD and IVM were extracted and analyzed using the Hydrophobic/philic Surfaces Panel. The hydrophobic and hydrophilic surface area of RBD was analyzed with the cut-off particular potential value (isovalue) of − 6 kcal/mol for hydrophilic regions and − 0.5 kcal/mol for hydrophobic regions. The trajectory file was imported into Visual Molecular Dynamics (VMD) software to calculate the solvent accessible surface area (SASA) and the radius of gyration (Rg) of the RBD protein in the different systems to study the protein stability over time^50^. Principal component analysis (PCA) using the pairwise distance method was performed on the RBD protein Cα to study the system motion during the MD simulation. The python package MDTraj was used to align the trajectory snapshots to the initial frame and construct the eigenvectors^51^.

## Results and discussion

### Binding kinetics of ivermectin

**Figure 1:**
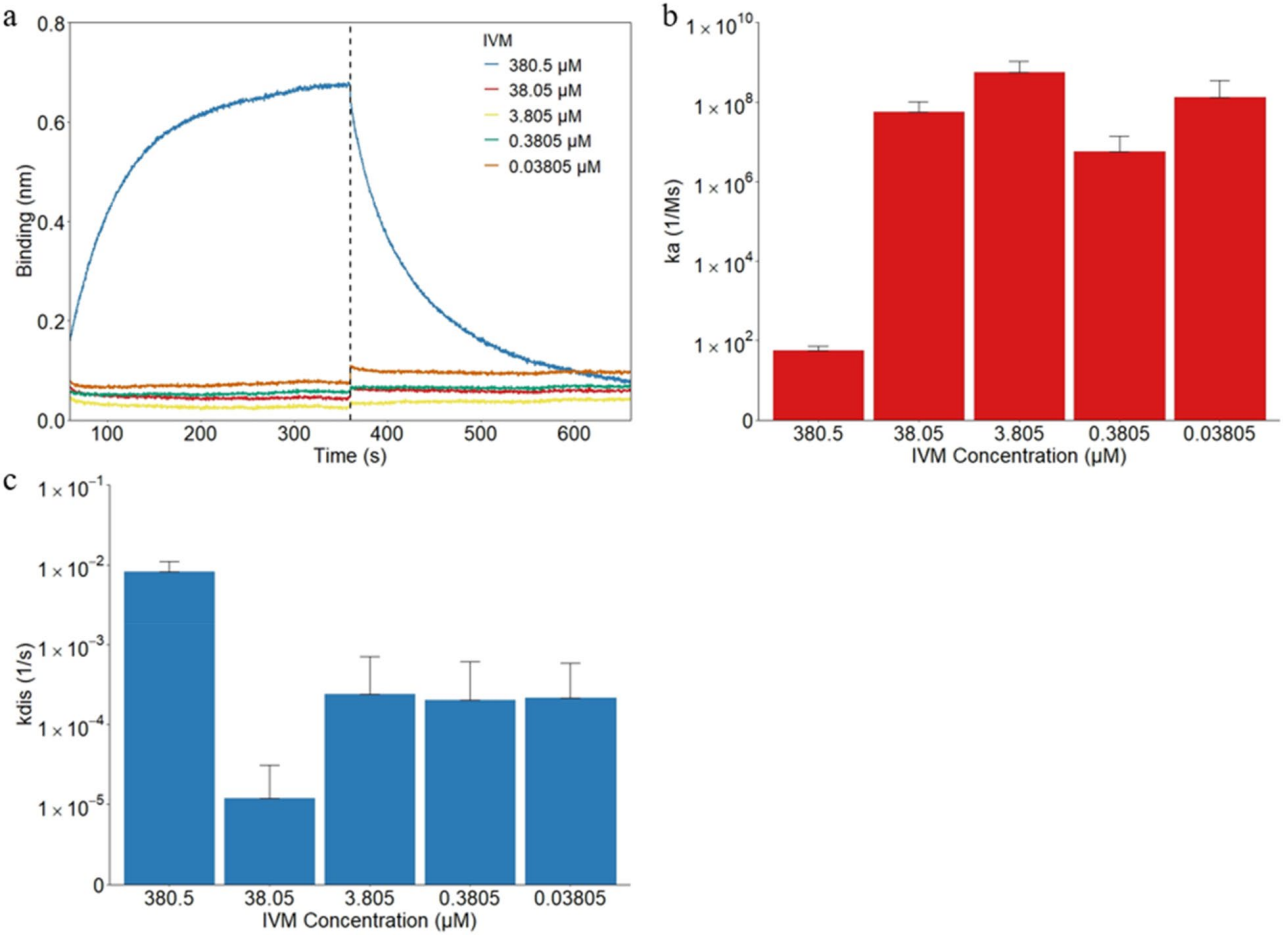
The binding kinetics for IVM only attach to and detach from the hydrophobic APS sensors. (**a**) The binding curves of IVM were generated using the basic kinetic analysis of IVM only at 380.5 μM, 38.05 μM, 3.805 μM, 0.3805 μM, 0.03805 μM. The vertical dotted line indicates the transition from the association step to the dissociation step. In the same order of the three groups, the association constant ka is shown in (**b**). The dissociation constant kdis is shown similarly in (**c**).

The Octet system determined the molar concentrations for IVM at the 1:1, 1:10, 1:100, 1:1000, and 1:10,000 dilutions to be 380.5 μM, 38.05 μM, 3.805 μM, 0.3805 μM, and 0.03805 μM based on the molecular weight of 0.8751 kDa. The basic kinetics analysis was performed at the average body temperature of 37 °C. A higher binding was shown for the highest IVM concentration, in which the 380.5 μM IVM demonstrated dramatic association and dissociation, while the other concentrations displayed binding at a consistently low level (Fig. 1a). The stronger binding capability of IVM with the highest concentration tested resulted in an average binding layer of 0.646 nm, compared to those of the other concentrations ranging from 0.033 and 0.109 nm by the end of the 300 s association step. This binding occurred throughout the entire association step but at a quicker rate toward the beginning. A similar observation of higher binding with increasing IVM concentration was made by Nappi et al. when IVM was tested against purified heat shock proteins 27 (HSP27) using BLI^52^. Previous study of IVM for the inhibition of the epidermal growth factor receptor (EGFR) also reported similar binding trends but at a lower range^53^. HSP27, EGFR, and ACE2 receptors all contain hydrophobic regions of various sizes which could explain the similarities and differences seen in their interactions with IVM^54–56^. The average association constants (ka), which measures the rate of sample binding to the hydrophobic biosensor, are shown in Fig. 1b. The average ka of 380.5 μM, 38.05 μM, 3.805 μM, 0.3805 μM, and 0.03805 μM IVM were 5.71 × 10^1^ Ms^−1^, 5.75 × 10^7^ Ms^−1^, 5.60 × 10^8^ Ms^−1^, 5.62 × 10^6^ Ms^−1^, and 1.33 × 10^8^ Ms^−1^, respectively. The 380.5 μM concentration was found to associate with the APS biosensors much weaker. Figure 1b demonstrates significantly different ka among tested IVM concentrations with an increase of five to seven magnitudes between 380.5 μM and the other concentrations (*p* = 0.05). The average dissociation constants (kdis) were also tested and returned similar results. The kdis values demonstrate how easily the IVM separates from the biosensor surface, the opposite of association and the average kdis of 380.5 μM, 38.05 μM, 3.805 μM, 0.3805 μM, and 0.03805 μM IVM were 8.20 × 10^–3^ s^−1^, 1.20 × 10^–5^ s^−1^, 2.38 × 10^–4^ s^−1^, 2.04 × 10^–4^ s^−1^, and 2.16 × 10^–4^ s^−1^, respectively. Figure 1c shows that the 380.5 μM IVM concentration demonstrates a significantly larger dissociation than all other concentrations (*p* < 0.0001). This difference is in line with what is seen in Fig. 1a. In the other studies mentioned above, the various concentrations of IVM ranging from 3.13 to 100 μM combined with their respective proteins demonstrated a binding curve that more closely resembles that of the 380.5 μM concentration^52^. Finally, the average equilibrium dissociation constants (KD) are determined by the system based on the ka and kdis values. For the IVM concentrations in decreasing concentration, the KD values were found to be 1.61 × 10^–4^ M, 1.21 × 10^–12^ M, 7.51 × 10^–11^ M, 1.86 × 10^–10^ M, and 4.81 × 10^–11^ M, respectively. The combination of the lowered association and increased dissociation of the 380.5 μM concentration leads to a much higher KD value, in the micromolar range, compared to the other concentrations in the picomolar range. While there is a large decrease in the KD values between the 380.5 and 38.05 μM concentrations (*p* = 0.0003), the trend begins to slowly increase again for the decreasing IVM concentrations. KD is inversely related to the affinity, and the higher the affinity the stronger the attraction between the drug and receptor. There are various uses for drugs varying within the micromolar to picomolar range and larger affinities do not always mean a more successful drug. For example, a study from 1984 found that the micromolar over nanomolar affinity of benzodiazepine behaved as a Ca^2+^ channel antagonist^57^.

### Binding kinetics of RBD

**Figure 2:**
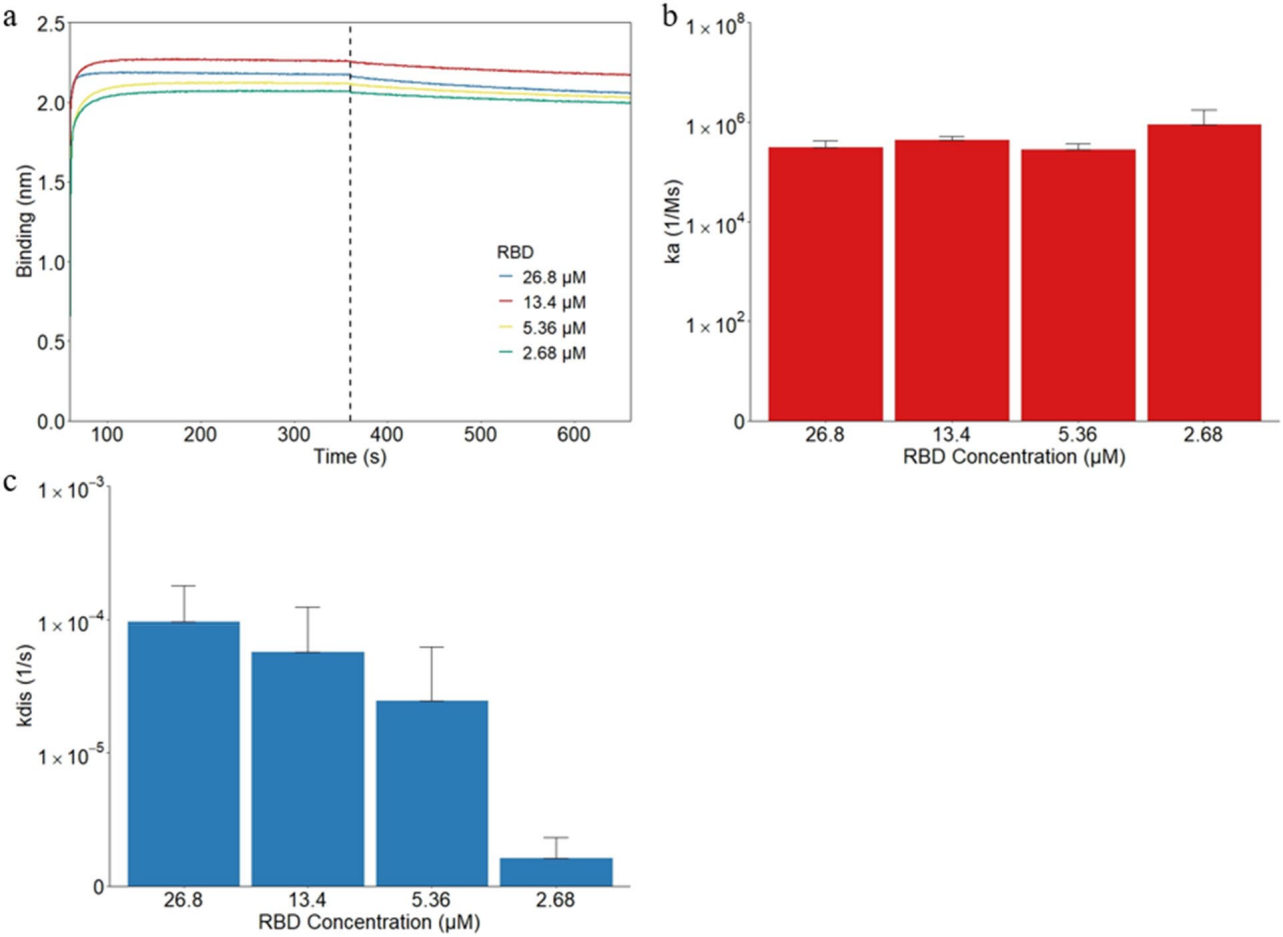
The binding kinetics for RBD only attach to and detach from the hydrophobic APS sensors. (**a**) The binding curves of IVM were generated using the basic kinetic analysis of RBD only at 26.8 μM, 13.4 μM, 5.36 μM, and 2.68 μM. The vertical dotted line indicates the transition from the association step to the dissociation step. In the same order as the three groups, the association constant ka is shown in (**b**). The dissociation constant kdis is shown similarly in (**c**).

The 1:1, 1:2, 1:5, and 1:10 dilutions of the original RBD were determined to have respective concentrations of 26.8 μM, 13.4 μM, 5.36 μM, and 2.68 μM based on the molecular weight of 58 kDa. The basic kinetics test was repeated with the RBD samples to determine a baseline on how RBD at these concentrations interacts with the hydrophobic sensors. At the beginning of the association step, all samples undergo significant binding before leveling out. By the end of the association step the average binding layers were found to be 2.170 nm, 2.256 nm, 2.115 nm, and 2.066 nm for 26.8 μM, 13.4 μM, 5.36 μM, and 2.68 μM RBD, respectively (Fig. 2a). This level of binding was overall much thicker than that of IVM seen in Fig. 1a, and stayed relatively unchanged over the testing period. The similarities in the binding curves are illustrated in Fig. 2b where the average ka values for 26.8 μM, 13.4 μM, 5.36 μM, and 2.68 μM RBD were found to be 3.10 × 10^5^ Ms^−1^, 4.41 × 10^5^ Ms^−1^, 2.80 × 10^5^ Ms^−1^, and 8.82 × 10^5^ Ms^−1^, respectively. The differences in association with varying RBD concentrations were not statistically significant (*p* = 0.124). On the other hand, a general decrease in dissociation with decreasing RBD concentration was found (*p* = 0.027). The corresponding kdis values for 26.8 μM, 13.4 μM, 5.36 μM, and 2.68 μM RBD were 9.65 × 10^–5^ s^−1^, 5.71 × 10^–5^ s^−1^, 2.45 × 10^–5^ s^−1^, and 1.00 × 10^–6^ s^−1^, respectively (Fig. 2c). Due to the similar values for the association values, the magnitude of the KD values was determined by the decreasing dissociation values with decreasing RBD concentrations (*p* = 0.023). Accordingly, average KD values were determined to be 3.78 × 10^–10^ M, 1.54 × 10^–10^ M, 9.33 × 10^–11^ M, and 2.85 × 10^–12^ M. Previous study performed on SARS-CoV RBD and hACE2 receptors used concentration ranges between 1.85 nM to 1.67 µM, overall smaller than the concentrations used in this experiment^58–60^. This difference is primarily seen in the thickness of the binding level which is much smaller than what is seen in this experiment. There is a consistent increase in binding demonstrated in all samples with increasing RBD concentration, the same general trend seen in this experiment. Additionally, the association rates are very similar with all results on the same magnitude of 10^5^ Ms^−1^. However, the dissociation values were consistently higher than the values seen in this experiment as they were consistently in the magnitude of 10^–3^ s^−1^. This is most likely due to the fact that the hACE2 was used instead of APS biosensors and other differences in the experimental setup. Due to the increased dissociation rates found in the studies, the KD values were also decreased to the nanomolar range. Overall, our findings are consistent with these studies with some differences due to the experimental setup.

### Binding kinetics of ivermectin and RBD combined

**Figure 3:**
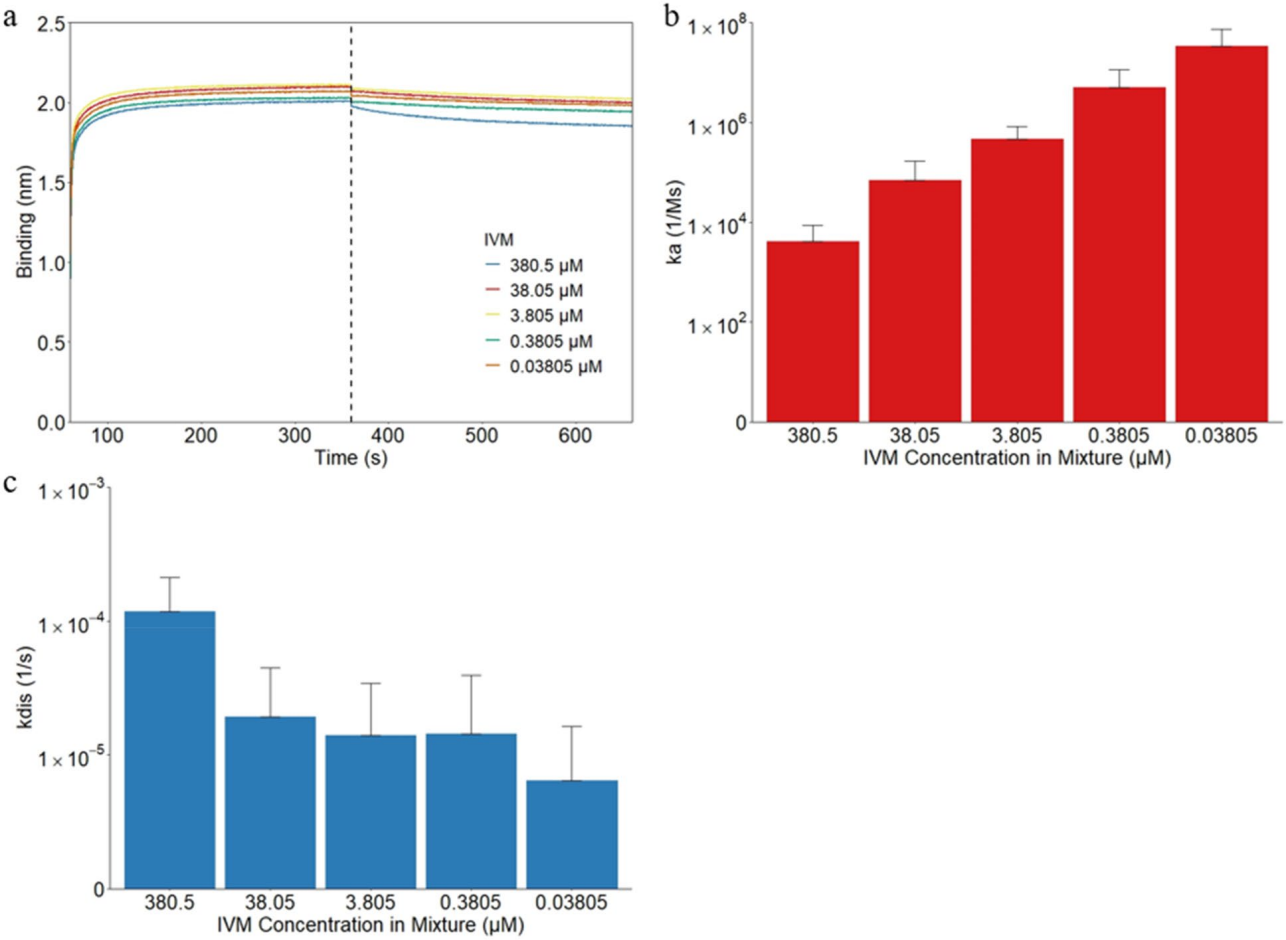
The binding kinetics for IVM and RBD combination attachment to and from the hydrophobic APS sensors. (**a**) The binding curves of IVM were generated using the basic kinetic analysis of 80.5 μM, 38.05 μM, 3.805 μM, 0.3805 μM, and 0.03805 μM IVM combined with 5.36 μM RBD. The vertical dotted line indicates the transition from the association step to the dissociation step. In the same order as the three groups, the association constant ka is shown in (**b**). The dissociation constant kdis is shown similarly in (**c**).

To observe the effect of IVM on the binding behaviors between RBD and the APS sensors, the basic kinetics test was performed with a 50/50 combination of the two. RBD at 5.36 μM concentration was used to mix with each of the IVM concentrations. Comparing Figs. 2a and 3a, it can be seen that overall the binding layers were slightly lower when the RBD was combined with IVM versus when it was RBD alone but still noticeably higher than IVM by itself. The average binding layers at the end of the association stage were 1.976 nm, 2.069 nm, 2.085 nm, 2.002 nm, and 2.039 nm for the 380.5 μM, 38.05 μM, 3.805 μM, 0.3805 μM, and 0.03805 μM IVM combined with 5.36 μM RBD, respectively. As seen in Fig. 3b, there is a consistent increase in association, with corresponding decreasing IVM concentrations (*p* = 0.0005). This trend is in line with what is seen in the IVM-only trials but demonstrates a more consistent increase. The ka values were found to be 4.12 × 10^3^ Ms^−1^, 6.90 × 10^4^ Ms^−1^, 4.74 × 10^5^ Ms^−1^, 5.03 × 10^6^ Ms^−1^, and 3.47 × 10^7^ Ms^−1^ for the decreasing IVM concentrations combined with RBD. Compared to the RBD-only results the combination with the 3.805 μM IVM provides a minimal effect on the association between RBD and the APS biosensor. However, the smaller concentrations demonstrate an increase in association while the larger concentrations show a decrease. One way to reduce the efficacy of viral replication is through competitive inhibition that defers the association of the virus to the body^61,62^. This negative cooperativity is only seen at the higher concentrations 38.05 μM and 380.5 μM. For the concentrations lower than 3.805 μM positive cooperativity is found where IVM aids in the binding of RBD and the hydrophobic sensor. The effects of the concentration of IVM on the dissociation rate of RBD can be clearly seen in Fig. 3c by the significantly larger kdis values for the 380.5 μM concentration that matches that seen in Fig. 1c. The kdis values were 1.18 × 10^–4^ s^−1^, 1.92 × 10^–5^ s^−1^, 1.40 × 10^–5^ s^−1^, 1.42 × 10^–5^ s^−1^, and 6.30 × 10^–6^ s^−1^, for decreasing IVM concentrations. The values for the 0.3805 μM, 3.805 μM, and 38.05 μM concentrations were close together and relatively unchanged from that of RBD only, but there was an overall decreasing trend for kdis values with decreasing IVM concentrations (*p* < 0.0001). Similar to the association values, the desired effect of increased dissociation is only found at the highest tested concentration, with opposite effects occurring at the smallest concentration. Compared to the 5.36 μM RBD-only sample that was found to have an average KD value of 9.33 × 10^–11^ M, there were split results with minimal effect found at the 3.805 μM combination. The KD values for decreasing IVM concentrations were 5.12 × 10^–8^ M, 3.90 × 10^–10^ M, 5.12 × 10^–11^ M, 7.81 × 10^–12^ M, and 1.13 × 10^–12^ M. Once again the 380.5 μM IVM concentration is in a different magnitude from the other concentrations, in the nanomolar range while the others were in the picomolar range. Overall, was a decreasing trend in KD values, and therefore increasing affinity, as the IVM concentrations decrease. The affinity of RBD to the hydrophobic sensor is both reduced and increased in a dose-dependent manner with IVM. Only at approximately 100 and 1,000 times the currently approved dosage are the desired effects found, which is in line with the findings of previous studies that the current dosage, and even 3 times higher, do not seem to have much of an effect^26,63^. In one study that tested IVM at 1200 μg/kg, 6 times the approved dosage, there was no significant reduction in viral load but more participants dropped out due to the tolerability of the high dosage and did not recommend additional trials at such a high level be performed^64^. While IVM is presumed to inhibit the nuclear translocation of viral proteins, clinical efficacy has not yet been proven^65,66^.

**Figure 4:**
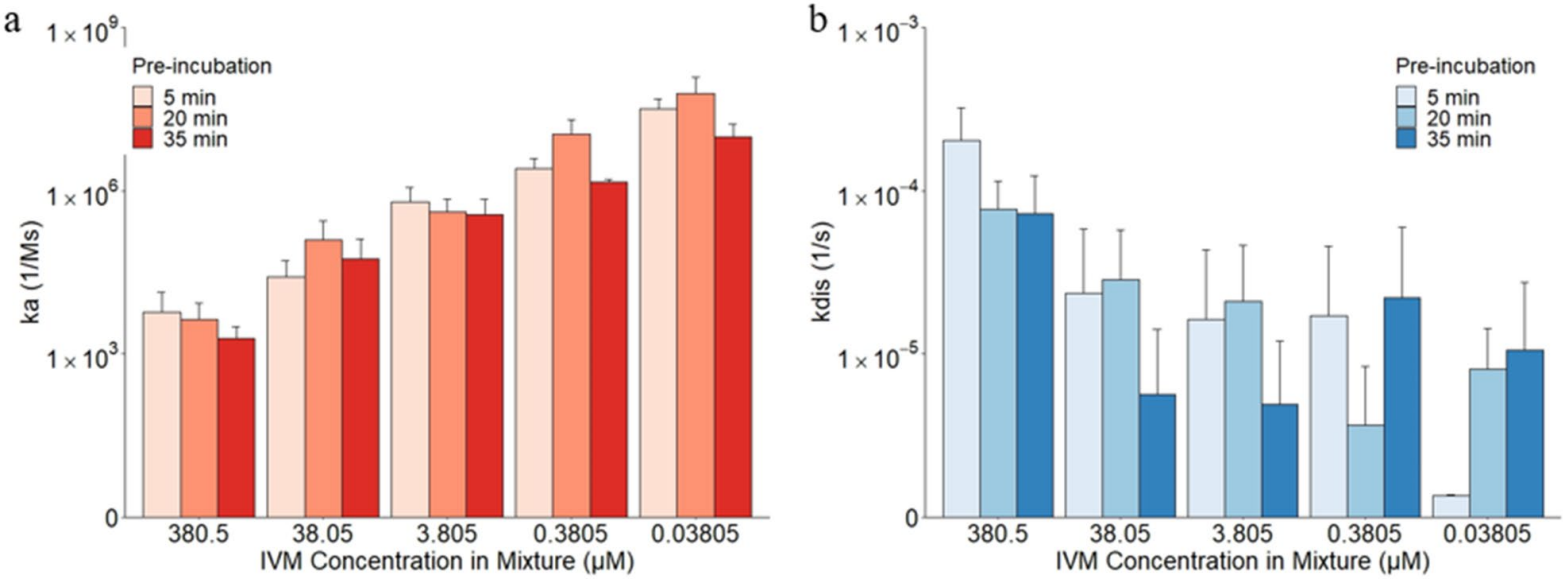
The effects of pre-incubation time on the attachment and detachment of the IVM and RBD complex to and from the hydrophobic APS sensors. (**a**) The association constant ka and (**b**) the dissociation constant kdis of 5.36 μM RBD and 380.5 μM, 38.05 μM, 3.805 μM, 0.3805 μM, and 0.03805 μM IVM combined with 5 min, 20 min, and 35 min pre-incubation.

The effect of IVM over an approximate 35-min pre-incubation time frame was determined using binding kinetics. Based on the pre-incubation time of each sample, the combination trial results were split into the following three groups: 5 min, 20 min, and 35 min. At the end of the association step the binding layers were between 1.842 nm and 2.175 nm for all concentrations and times (Supplementary Fig. 1). The statistical significance of the effect of IVM concentration or pre-incubation time on ka, kdis, and KD were obtained using ANOVA tests and listed in Supplementary Table 1. A general increasing trend of association for decreasing IVM concentrations was observed in the time trials (*p* = 0.0013–0.098). While, nominal changes in the association values of the IVM and RBD complex to the hydrophobic APS sensors over time for each given concentration (*p* = 0.144–0.67), as seen in Fig. 4. There was a correlation between decreasing IVM concentration and decreasing kdis values (*p* = 0.008–0.0858). The low kdis values for the 0.03805 μM IVM with 5-min pre-incubation could be explained by the hydrophobicity analysis from the MD simulation in the following sections. With a small amount of IVM, the hydrophobic residues that were initially embedded in the RBD were attracted outwards to interact with the IVM, which in turn increased the hydrophobic area on the RBD surface. The hydrophobic regions were then occupied by the IVM molecules when IVM concentration was sufficient, leading to a higher kdis value as shown with the higher IVM concentration results. When the IVM concentration was low and depleted, however, RBD binding to the hydrophobic sensor became stronger which resulted in a low kdis value. Additionally, no discernable trend was observed for the dissociation values of each IVM concentration combination with RBD over the time frame (*p* = 0.144–0.699). Despite the fluctuations of the association and dissociation values, KD remained relatively unchanged throughout the testing process (*p* = 0.291–0.798). Each of the average KD time trial results for the individual IVM concentration is within the same order of magnitude as that of the combined combination trials and following the same increasing trend with decreasing IVM concentration (*p* = 0.0036–0.015). Therefore, pre-incubation time did not have a statistically significant effect on ka, kdis, or KD at all tested IVM concentrations. One potential reason for the minimal changes found across the span of this experiment is the relatively short incubation period of 5–35 min compared to 46 h for the in vitro study and 0 to 24 h in animal studies^13,29,30^. However, in comparison to MD simulations of the interactions that are typically performed in the ns range, it is much longer^8,36^. It is important to consider the kinetics, interactions, and effects of IVM over a wide scope of time differences to ensure efficacy and safety when considering repurposing IVM for use with COVID-19.

### Protein–ligand docking verification

**Figure 5:**
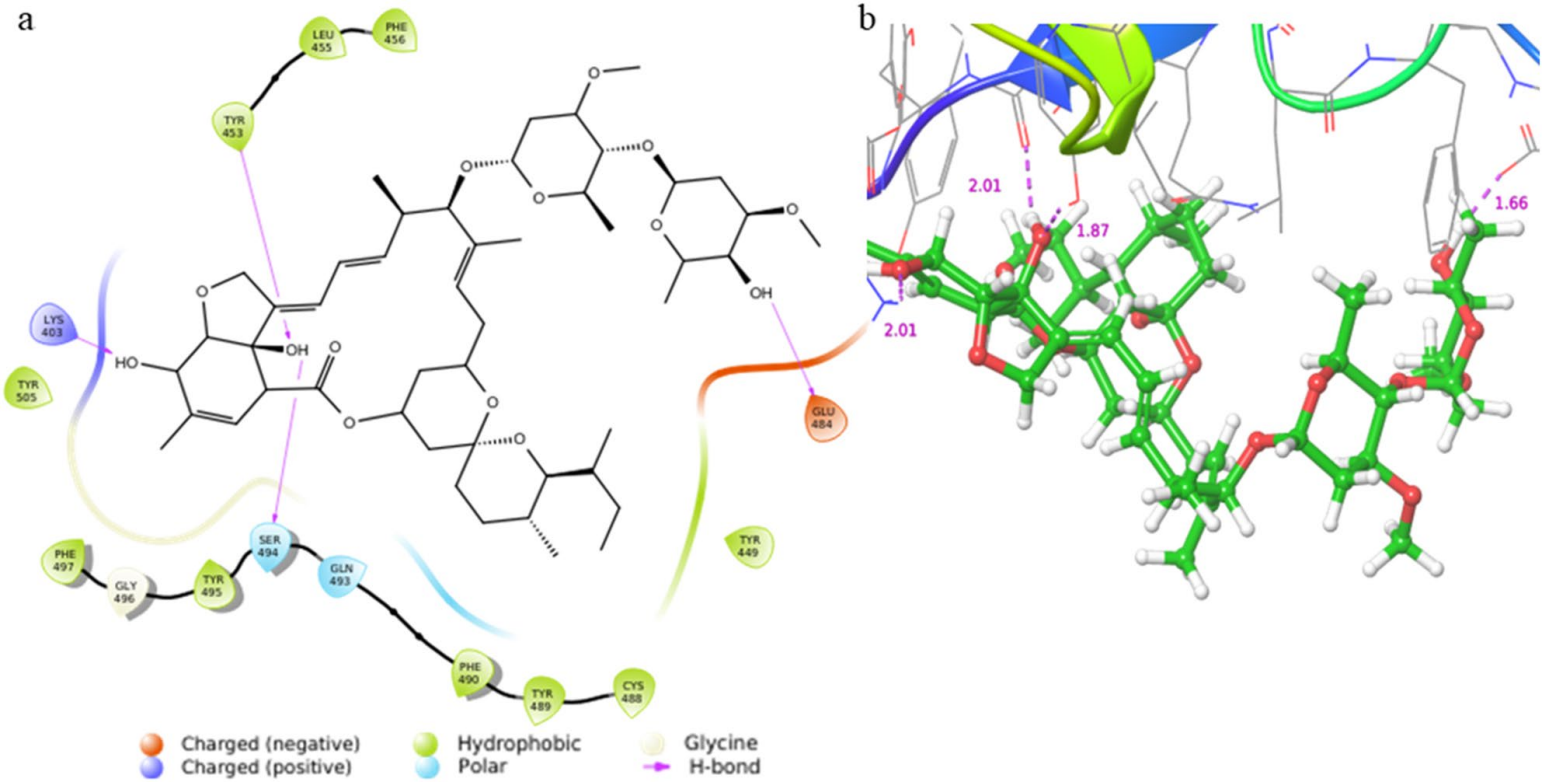
The docking pose of IVM with SARS-CoV-2 RBD in (**a**) 2D and (**b**) 3D interaction diagrams. The hydrogen bonds formed between IVM and RBD residues from the left to right in the 3D interaction diagram are SER 494, LYS 403, TYR 453, and GLU 484, and the corresponding bond lengths are shown in purple. The RBD residues that interacted with the IVM are labeled.

The highest binding affinity of IVM with RBD was − 4.73 kcal/mol. The docking pose of the IVM to the RBD at the binding site was shown in 2D and 3D space. Multiple non-covalent bonds were formed between the IVM and RBD, including four hydrogen bonds at residues LYS 403, TYR 453, GLU 484, and SER 494, hydrophobic interactions at TYR 449, CYS 488, TYR 489, PHE 490, TYR 495, PHE 497, TYR 505, PHE 456, and LEU 455, and one polar bond at GLN 493 (Fig. 5a). The distances of the hydrogen bonds were 1.66 Å, 2.01 Å, 2.01 Å, and 1.87 Å at residues GLU 484, SER 494, LYS 403, and TYR 453, respectively (Fig. 5b). Our results agree with the study of Saha and Raihan^67^ that LEU 455, a residue that favors interaction with human ACE2, can bind to IVM. The binding between RBD and IVM is largely regulated by hydrophobic interactions, similar to that of the human ACE2-IVM complex that was reported in a previous study^68^. Many of the residues—GLN 493, TYR 505, TYR 449, TYR 489, PHE 456, TYR 495, and LEU 455—that were found to regulate the binding between RBD and IVM were previously identified as hotspots in interactions between RBD and ACE2, which made IVM a candidate for drug repurposing for COVID-19^69^.

### Molecular dynamics

**Figure 6:**
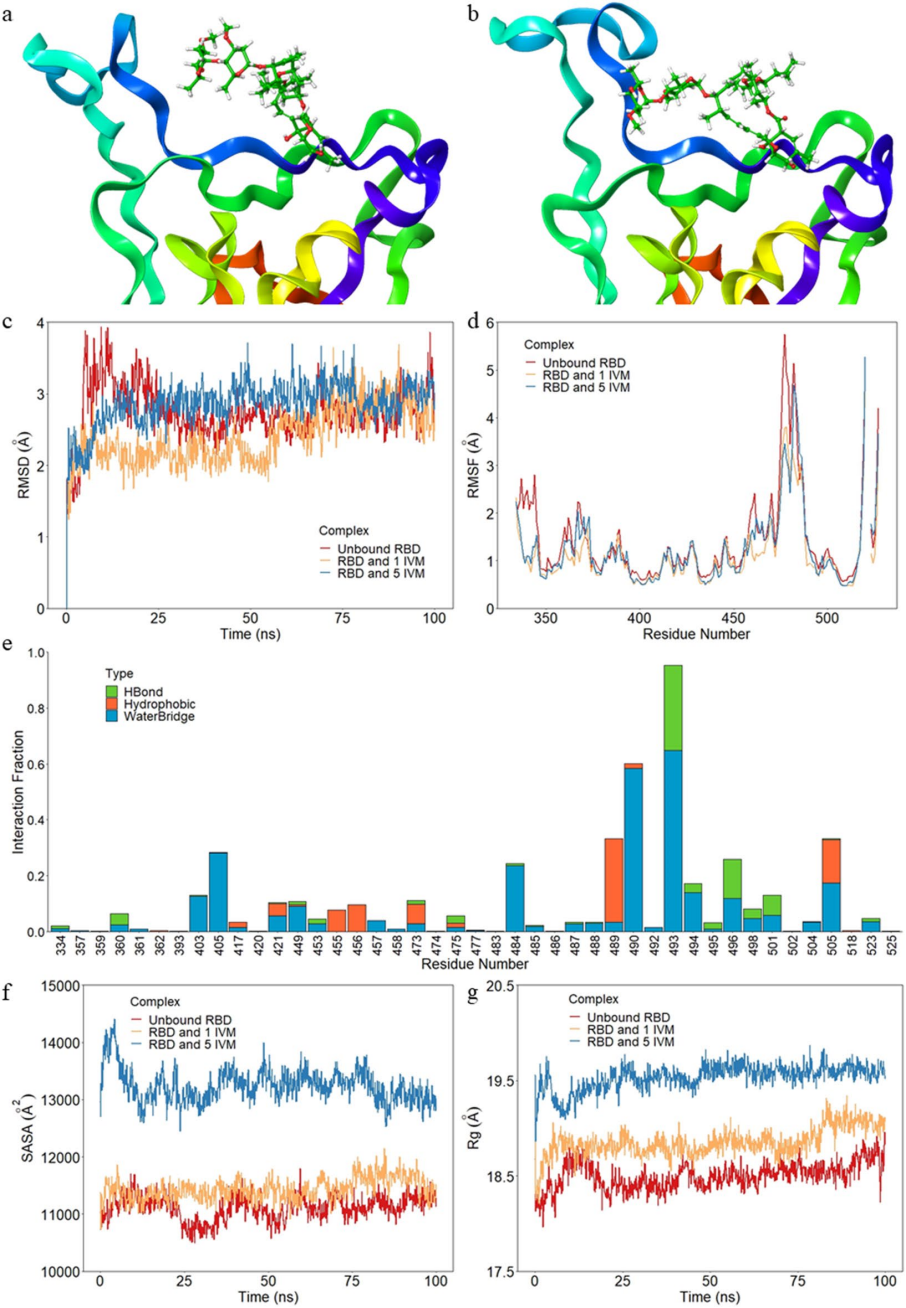
Structural properties of the protein–ligand complexes during MD simulation. (**a**) Initial and (**b**) final snapshots of RBD protein bound with IVM. (**c**) The RBD protein Cα RMSD values over time for the average displacement change in the unbound RBD (red), RBD with one IVM molecule (yellow), and RBD with five IVM molecules (blue). (**d**) Residue-based protein Cα RMSF over the trajectory for local fluctuation along the protein chain in the unbound RBD (red), RBD with one IVM molecule (yellow), and RBD with five IVM molecules (blue). (**e**) Fraction of RBD interactions with the IVM molecule over the trajectory categorized into hydrogen bonds (green), hydrophobic interactions (orange), and water bridges (blue). (**f**) SASA and (**g**) Rg of the RBD protein over time in the unbound RBD (red), RBD with one IVM molecule (yellow), and RBD with five IVM molecules (blue).

All-atom molecular dynamics in an explicit solvent model with a total length of 100 ns was performed on three protein–ligand complexes to further understand the interaction between IVM and RBD. In order to determine which water model to be used in the full-length simulation, short MD simulations of 10 ns were performed on the docked RBD and IVM complex with three widely exploited explicit water models—SPC, SPC/E, and TIP3P. The protein Cα RMSD values with the three solvent models were compared and all three models showed similar RMSD values and fluctuation throughout the 10 ns simulations (Supplementary Fig. 2). Therefore, the TIP3P model was chosen for the full-scale 100 ns MD simulations as the water molecules in the TIP3P model have rigid geometry that closely matches the actual situation^70^. 0.1 M explicit Na^+^ and Cl^−^ ions were added in the solvent model and two additional Cl^−^ ions were added to neutralize the system. The MD simulations were performed on three complexes—unbound RBD, RBD with one IVM, and RBD with five IVM molecules—under a temperature of 300 K and pressure of 1 atm for 100 ns. The unbound RBD served as a control to compare for any structural changes of the protein that was induced by IVM. The initial and final snapshots of the superimposed structures of the RBD protein bound with IVM showed that IVM can bind and stay closely in the active site of the RBD protein (Fig. 6a,b). The binding energy between the RBD protein and the IVM was calculated using the MM-GBSA method from the last 40 ns of the MD simulation trajectory. The overall average binding free energy between the RBD protein and the IVM was − 50.20 ± 8.32 kcal/mol, which was considered as high binding energy by other studies and supported the protein–ligand docking result of high binding affinity between the RBD protein and the IVM^71–73^. The RBD protein Cα RMSD values of the three complexes were monitored throughout the MD simulations and analyzed to show the stability of protein structure (Fig. 6c). All simulations were performed in three replicates and the other two data sets of each system were included in Supplementary Fig. 3–5. The average RMSD value of the unbounded RBD protein, RBD protein with one IVM molecule, and RBD protein with five IVM molecules were 2.77 ± 0.44 Å, 2.48 ± 0.41 Å, and 2.74 ± 0.28 Å, respectively, and all the MD simulations were considered stable after 60 ns. In the unbound RBD, the RMSD value increased during the first 25 ns and reached a high value of 4 Å, then it reached equilibrium around 2.7 Å with small fluctuation until the end of the simulation. The complex of RBD and one IVM molecule maintained a stable RMSD of 2.2 Å for the first half of the simulation, then the RMSD value increased slightly after 55 ns up to around 3 Å. For RBD with five IVM molecules, the RMSD value increased during the first 20 ns and remained stable at around 3 Å for the rest of the simulation. Comparing the two complexes with IVM to the control, it was shown that the RMSD values of the three systems overlapped during the second 50 ns of the simulations. This indicates that the protein structure remained relatively the same during the simulations. However, RBD with five IVM took shorter time to stabilize than with one IVM molecule, suggesting that the increased IVM concentration in the system was beneficial for the protein stability. The protein Cα RMSF was calculated over the trajectory to investigate the structural fluctuation in terms of residues. The two protein–ligand complexes shared the same RMSF features as the unbound RBD alone, with residues 475 to 486 fluctuating the most during the simulations (Fig. 6d). The location and fraction of RBD interaction with the IVM molecule during the simulation were categorized into three interaction types—hydrogen bonds (H bonds), hydrophobic interaction, and water bridge (Fig. 6e). During the 100 ns simulation, the interaction between IVM and residue 493 was maintained the entire time and with residue 490 for 60% of the time. Other residues that maintained an interaction over 20% of the time were residues 405, 484, 489, 496, and 505. Residues 484 and 493 were shown to play critical roles in the RBD and human ACE2 binding^39^. The strong contact and stable interaction between these two residues and IVM indicated that IVM might be able to prevent and interrupt the RBD-ACE2 binding, hence that several published in silico studies reported IVM as a potential treatment for COVID-19^67,68,74,75^. However, more experimental evidence is still required to further investigate the effect of IVM in preventing or treating SARS-CoV-2, and this study attempts to fill this gap.

To further study the structural properties of the RBD protein in the systems with or without IVM molecules, post-MD analysis such as SASA and Rg were performed. SASA of a protein is defined as the protein surface area that is accessible to the solvent and is a measurement of protein stability^76^. The average SASA values for the unbounded RBD protein, RBD protein with one IVM, and RBD protein with five IVM were 11,067.36 ± 210.72 Å^2^, 11,471.19 ± 216.84 Å^2^, and 13,085.14 ± 324.78 Å^2^, respectively. There was no major change of SASA values between the unbound RBD protein and the RBD protein with one IVM molecule, however, the SASA of the RBD protein with five IVM molecules was higher than the other two systems (Fig. 6f). The RBD protein with five IVM molecules having higher SASA therefore indicated a less stable overall protein structure compared to the unbound RBD protein and the RBD protein with only one IVM, while the RBD protein in the other two systems exhibited no major difference in protein stability. The Rg measures the compactness of the protein structure and is another indicator for protein stability. The average Rg values for the unbounded RBD protein, RBD protein with one IVM, and RBD protein with five IVM were 18.50 ± 0.14 Å, 18.74 ± 0.17 Å, and 19.52 ± 0.17 Å, respectively. Similar to the SASA, the RBD protein in the complex with five IVM molecules exhibited higher Rg compared to the other two systems, while no major difference in Rg was observed between the unbound RBD protein and the RBD protein with one IVM molecule. Although the RBD with five IVM molecules had an overall higher Rg, the Rg value showed low variation and stayed steady after 50 ns of the simulation, suggesting a still relatively stable protein structure (Fig. 6g).

**Figure 7:**
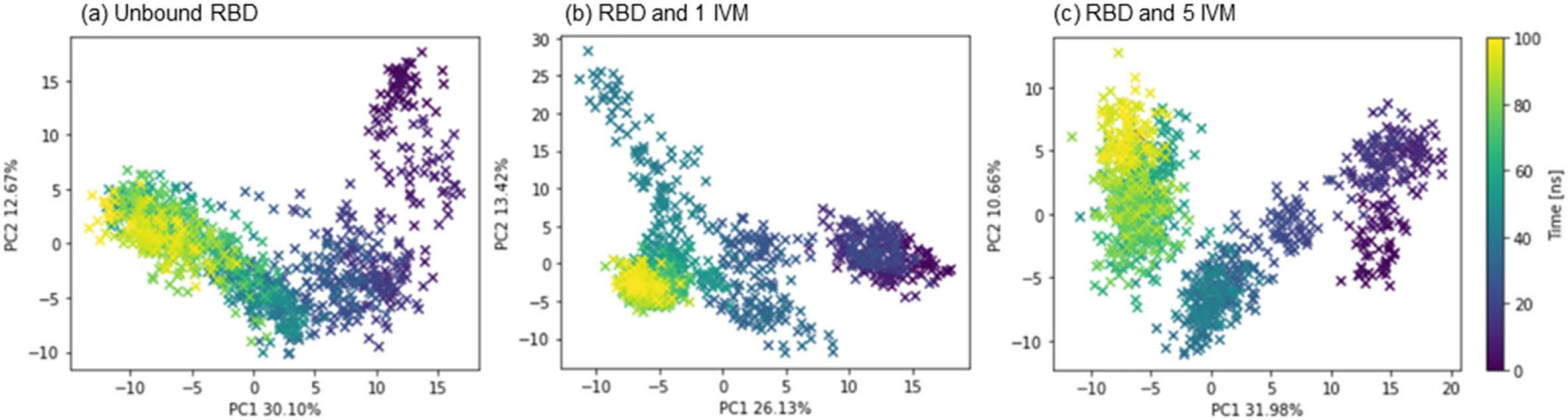
Pairwise distance PCA on the RBD protein Cα from the MD simulation trajectory. The first two eigenvectors PC1 and PC2 were used to construct the PCA in (**a**) unbound RBD, (**b**) RBD and one IVM molecule, and (**c**) RBD and five IVM molecules.

Pairwise distance PCA was performed over the 100 ns trajectory to further study the motion of the protein system in the MD simulations (Fig. 7a–c). PCA has been used to describe the total fluctuations of systems during MD simulation with drastic dimensionality reduction^76–78^. The first two eigenvectors PC1 and PC2 accounted for ~ 40% of the total variance in the three systems—unbound RBD protein, RBD protein with one IVM molecule, and RBD protein with five IVM molecules—and were used to construct the PCA. The PCA plots of all the three systems demonstrated smooth and consecutive changes of the data points with no outliers, with the clustering of data points occurring after 60 ns (light green and yellow points) suggesting stabilization of the protein structure. The concentrated cluster distribution in the last 40 ns of the RBD protein with one IVM was an indicator of reduced protein flexibility possibly due to ligand binding, which were in agreement with the other parameters such as RMSD and SASA.

**Figure 8:**
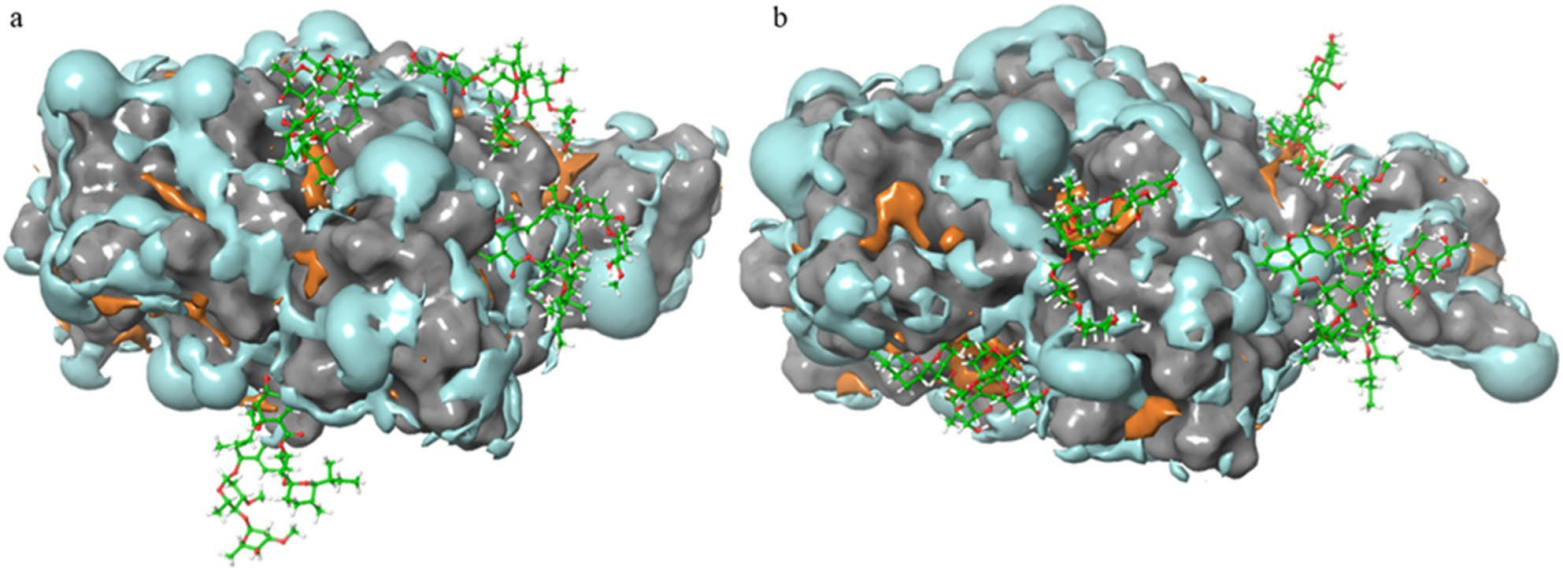
Hydrophobic (orange) and hydrophilic (cyan) surface of RBD interacting with 5 IVM molecules (green sticks) in the (**a**) initial and (**b**) final frame of MD simulation.

The initial and final frame of the MD simulation of RBD with five IVM molecules were analyzed for the protein surface hydrophobicity. The IVM molecules moved towards RBD and attached to the hydrophobic RBD surface at the end of the simulation (Fig. 8a,b). The area of the hydrophobic RBD surface increased from 846.5 Å^2^ at the initial frame to 1143.5 Å^2^ at the final frame when interacting with IVM molecules. It was hypothesized that the increased hydrophobicity of RBD was due to IVM attracting the embedded hydrophobic residues to come out to interact with the ligand. By including one and more than one (five) IVM molecules in the system, we aimed to study the effect of the increasing IVM concentration on the protein stability and surface properties which might explain the different kinetic parameters that were experimentally observed. A maximum of five IVM molecules were tested in this study due to the constraints of time and resources. With more IVM in the solvent system, it is likely that a majority of the RBD hydrophobic area would be occupied by the ligands, which in turn reduces the binding ability of the RBD to the APS biosensor. This could explain why increasing IVM concentration led to decreasing rate of association of RBD in the experiment. It is important to note that, with increasing numbers of IVM molecules in the in silico system, the equivalent dosage in human body will exceed the established dosage limit of 150–200 μg/kg that was proved to have adverse effects for human^33^.

## Conclusions

The binding behaviors are an instrumental aspect when developing effective treatment or prevention methods for the virus. This study utilized BLI technology to determine the binding kinetics and affinity of various IVM concentrations to a hydrophobic biosensor and their effect when combined with RBD. Negative cooperativity, where IVM slows or prevents RBD from binding to the biosensor, was only found at 38.05 μM and especially at 380.5 μM, approximately 100 and 1,000 times the approved IVM dosage. These same concentrations were able to weaken the affinity levels between RBD and the hydrophobic biosensor as desired, while the lower concentrations had a minimal effect or strengthened it. Only nominal effects on the attachment and detachment of the IVM and RBD complex to and from the hydrophobic APS sensors were seen due to changes in pre-incubation time. The strong binding capability between IVM and RBD was verified through in silico molecular docking. The RBD residues that interacted with IVM were found to be hotspot residues in the RBD-ACE2 complex, showing that IVM might be able to interrupt the SARS-CoV-2 infection in human cells which makes IVM a potential prevention and treatment of COVID-19. MD simulation of the unbound RBD and RBD with one and five IVM molecules demonstrated good stability of the complex structure over the 100 ns simulation time. Comparison analysis between the three complexes further revealed that higher IVM concentration triggers an increase in the hydrophobic surface area of RBD, which changes its binding ability. Overall, based on our results, it is not recommended that IVM, at the currently approved dosage, be used as a preventative or treatment method. The established dosage used in this experiment is based on oral administration. This provides the drug with the opportunity to disperse throughout the body, while COVID-19 on the other hand is centralized in the respiratory system. Other methods, such as nasal spray or nebulizer treatment, should be considered as they are better suited for direct delivery to the respiratory system. Potential future studies could investigate IVM on the different concentrations of RBD to mimic the varying viral loads. This change, in addition to more extended time trials, will allow a further understanding of the effects of IVM throughout the various stages of the viral infection.

### Supplementary Information

Supplementary Information.

## Data Availability

All data generated or analyzed during this study are included in this manuscript and its Supplementary Materials.

## References

[1] WHO coronavirus (COVID-19) dashboard. World Health Organization. https://covid19.who.int/

[2] Wu D (2020). The SARS-COV-2 outbreak: What we know. Int. J. Infect. Dis..

[3] Ciotti M (2020). The COVID-19 pandemic. Crit. Rev. Clin. Lab. Sci..

[4] Cabrera Martimbianco AL (2021). Frequency, signs and symptoms, and criteria adopted for long Covid-19: A systematic review. Int. J. Clin. Pract..

[5] Shabir, O. What is a receptor-binding domain (RBD)? News. https://www.news-medical.net/health/What-is-a-Receptor-Binding-Domain-(RBD).aspx

[6] Sharma G (2022). Effect of an inhibitor on the ACE2-receptor-binding domain of SARS-COV-2. J. Chem. Inf. Model..

[7] de Vries, L. Antibody mining in SARS-COV-2 spike-immunized rhesus macaques. Utrecht University Student Theses Repository Home. https://studenttheses.uu.nl/handle/20.500.12932/43585. Accessed May 22, 2023.

[8] Celik I (2021). Interactions of the receptor binding domain of SARS-COV-2 variants with HACE2: Insights from molecular docking analysis and molecular dynamic simulation. Biology.

[9] Eskandari V (2022). Repurposing the natural compounds as potential therapeutic agents for covid-19 based on the molecular docking study of the main protease and the receptor-binding domain of Spike protein. J. Mol. Model..

[10] Chen LL (2022). Impact of severe acute respiratory syndrome coronavirus 2 (SARS-CoV-2) variant-associated receptor binding domain (RBD) mutations on the susceptibility to serum antibodies elicited by coronavirus disease 2019 (COVID-19) infection or vaccination. Clin. Infect. Dis..

[11] Min L, Sun Q (2021). Antibodies and vaccines target RBD of SARS-CoV-2. Front. Mol. Biosci..

[12] Guan X (2023). Advances in SARS-CoV-2 receptor-binding domain-based COVID-19 vaccines. Expert Rev. Vaccines.

[13] Caly L (2020). The FDA-approved drug ivermectin inhibits the replication of SARS-CoV-2 in vitro. Antiviral Res..

[14] Jans DA, Wagstaff KM (2021). The broad spectrum host-directed agent ivermectin as an antiviral for SARS-COV-2?. Biochem. Biophys. Res. Commun..

[15] Miyamoto Y (2016). Importin α: A key molecule in nuclear transport and non-transport functions. J. Biochem..

[16] Delandre O (2022). Antiviral activity of repurposing ivermectin against a panel of 30 clinical SARS-COV-2 strains belonging to 14 variants. Pharmaceuticals.

[17] Zaidi AK, Dehgani-Mobaraki P (2022). The mechanisms of action of ivermectin against SARS-CoV-2-an extensive review. J. Antibiot..

[18] Santin AD (2021). Ivermectin: A multifaceted drug of Nobel prize-honoured distinction with indicated efficacy against a new global scourge, covid-19. New Microb. New Infect..

[19] Molento MB (2021). Ivermectin against COVID-19: The unprecedented consequences in Latin America. One Health.

[20] Yagisawa M (2021). Global trends in clinical studies of ivermectin in COVID-19. Jpn. J. Antibiot..

[21] de la Rocha C (2022). Ivermectin compared with placebo in the clinical course in Mexican patients with asymptomatic and mild COVID-19: A randomized clinical trial. BMC Infect. Dis..

[22] Alam MT (2020). Ivermectin as pre-exposure prophylaxis for COVID-19 among healthcare providers in a selected tertiary hospital in Dhaka—An observational study. Eur. J. Med. Health Sci..

[23] Kerr L (2022). Ivermectin prophylaxis used for COVID-19: A citywide, prospective, observational study of 223,128 subjects using propensity score matching. Cureus.

[24] Chaccour C (2021). The effect of early treatment with ivermectin on viral load, symptoms and humoral response in patients with non-severe COVID-19: A pilot, double-blind, placebo-controlled, randomized clinical trial. EClinicalMedicine.

[25] Schilling WHK (2023). Pharmacometrics of high-dose ivermectin in early COVID-19 from an open label, randomized, controlled adaptive platform trial (PLATCOV). eLife.

[26] Naggie S (2023). Effect of higher-dose ivermectin for 6 days versus placebo on time to sustained recovery in outpatients with COVID-19: A randomized clinical trial. JAMA.

[27] Ivermectin Nasal Spray for COVID19 Patients-Full Text View. ClinicalTrials.Gov, classic.clinicaltrials.gov/ct2/show/NCT04510233.

[28] Inhaled Ivermectin and COVID-19-Full Text View. ClinicalTrials.Gov, classic.clinicaltrials.gov/ct2/show/NCT04681053.

[29] Errecalde J (2021). Safety and pharmacokinetic assessments of a novel ivermectin nasal spray formulation in a pig model. J. Pharmaceut. Sci..

[30] Chaccour C (2020). Nebulized ivermectin for COVID-19 and other respiratory diseases, a proof of concept, dose-ranging study rats. Sci. Rep..

[31] Francés-Monerris A (2021). Microscopic interactions between ivermectin and key human and viral proteins involved in SARS-COV-2 infection. Phys. Chem. Chem. Phys..

[32] Maurya, D. K. A combination of ivermectin and doxycycline possibly blocks the viral entry and modulates the innate immune response in COVID-19 patients (2020). 10.26434/chemrxiv.12630539.v1

[33] National Institutes of Health. Ivermectin tablets USP, 3mg (ivermectin). U.S. National Library of Medicine (n.d.). https://dailymed.nlm.nih.gov/dailymed/fda/fdaDrugXsl.cfm?setid=847a1dd7-d65b-4a0e-a67d-d90392059dac&type=display

[34] Fryar CD (2021). Anthropometric reference data for children and adults: United States, 2015–2018. National Center for Health Statistics. Vital Health Stat..

[35] Concepcion J (2009). Label-free detection of biomolecular interactions using BioLayer interferometry for kinetic characterization. Combin. Chem. High Throughput Screen..

[36] Wang Y (2020). Enhanced receptor binding of SARS-COV-2 through networks of hydrogen-bonding and hydrophobic interactions. Proc. Natl. Acad. Sci..

[37] Tai W (2020). Characterization of the receptor-binding domain (RBD) of 2019 novel coronavirus: Implication for development of RBD protein as a viral attachment inhibitor and vaccine. Cell. Mol. Immunol..

[38] Zhang M (2022). Binding behavior of Spike protein and receptor binding domain of the SARS-COV-2 virus at different environmental conditions. Sci. Rep..

[39] Shang J (2020). Structural basis of receptor recognition by SARS-CoV-2. Nature.

[40] Berman HM (2000). The protein data bank. Nucleic Acids Res..

[41] Schrödinger. Schrödinger Suite 2018-4 Protein Preparation Wizard, Epik (Schrödinger, 2018).

[42] Prime, S. 40 (LLc, 2017).

[43] National Center for Biotechnology Information. PubChem Compound Summary for CID 6321424, Ivermectin B1a (2023). https://pubchem.ncbi.nlm.nih.gov/compound/Ivermectin-B1a

[44] Schrödinger Release. 2: LigPrep (Schrödinger, 2017).

[45] Schrödinger Release 2021-4: Glide (Schrödinger, LLC, 2021).

[46] Release, S. 1: Maestro (Schrödinger, LLC, 2017).

[47] Release S (2017). 4: Desmond Molecular Dynamics System.

[48] Roos K (2019). OPLS3e: Extending force field coverage for drug-like small molecules. J. Chem. Theory Comput..

[49] Li J (2011). The VSGB 2.0 model: A next generation energy model for high resolution protein structure modeling. Proteins.

[50] Humphrey W (1996). VMD: Visual molecular dynamics. J. Mol. Graph..

[51] McGibbon RT (2015). MDTraj: A modern open library for the analysis of molecular dynamics trajectories. Biophys. J..

[52] Nappi L (2020). Ivermectin inhibits HSP27 and potentiates efficacy of oncogene targeting in tumor models. J. Clin. Investig..

[53] Jiang L (2019). Ivermectin reverses the drug resistance in cancer cells through EGFR/ERK/Akt/NF-κB pathway. J. Exp. Clin. Cancer Res..

[54] Martin-Fernandez ML (2019). Structure and dynamics of the EGF receptor as revealed by experiments and simulations and its relevance to non-small cell lung cancer. Cells.

[55] Takeuchi S (2006). Analytical assays of human HSP27 and thermal-stress survival of *Escherichia coli* cells that overexpress it. Biochem. Biophys. Res. Commun..

[56] Prabakaran P (2004). A model of the ACE2 structure and function as a SARS-CoV receptor. Biochem. Biophys. Res. Commun..

[57] Taft WC, DeLorenzo RJ (1984). Micromolar-affinity benzodiazepine receptors regulate voltage-sensitive calcium channels in nerve terminal preparations. Proc. Natl. Acad. Sci. U. S. A..

[58] Yi C (2020). Key residues of the receptor binding motif in the spike protein of SARS-CoV-2 that interact with ACE2 and neutralizing antibodies. Cell. Mol. Immunol..

[59] Haycroft ER (2023). Antibody Fc-binding profiles and ACE2 affinity to SARS-CoV-2 RBD variants. Med. Microbiol. Immunol..

[60] Ou J (2022). ACE2-targeting antibody suppresses SARS-CoV-2 omicron and delta variants. Signal Transduct. Target. Ther..

[61] Yanai H (2020). Favipiravir: A possible pharmaceutical treatment for covid-19. J. Endocrinol. Metabol..

[62] Ullah SF (2022). An experimental framework for developing point-of-need biosensors: Connecting bio-layer interferometry and electrochemical impedance spectroscopy. Biosensors.

[63] Schmith VD (2020). The approved dose of ivermectin alone is not the ideal dose for the treatment of COVID-19. Clin. Pharmacol. Therap..

[64] Buonfrate D (2022). High-dose ivermectin for early treatment of COVID-19 (COVER study): A randomised, double-blind, multicentre, phase II, dose-finding, proof-of-concept clinical trial. Int. J. Antimicrob. Agents.

[65] Kinobe RT, Owens L (2021). A systematic review of experimental evidence for antiviral effects of ivermectin and an in silico analysis of Ivermectin’s possible mode of action against SARS-COV-2. Fundam. Clin. Pharmacol..

[66] Ragó Z (2023). Results of a systematic review and meta-analysis of early studies on ivermectin in SARS-COV-2 infection. GeroScience.

[67] Saha JK, Raihan MJ (2021). The binding mechanism of ivermectin and levosalbutamol with spike protein of SARS-CoV-2. Struct. Chem..

[68] Choudhury A (2021). Exploring the binding efficacy of ivermectin against the key proteins of SARS-CoV-2 pathogenesis: An in silico approach. Future Virol..

[69] B. Jawad *et al.* Key Interacting Residues between RBD of SARS-COV-2 and ACE2 Receptor: Combination of Molecular Dynamic Simulation and Density Functional Calculation (2021). 10.26434/chemrxiv.14582484 10.1021/acs.jcim.1c00560 34428371

[70] Mark P, Nilsson L (2001). Structure and dynamics of the TIP3P, SPC, and SPC/E water models at 298 K. J. Phys. Chem. A.

[71] Pirolli D (2023). Virtual screening and molecular dynamics simulations provide insight into repurposing drugs against SARS-CoV-2 variants Spike protein/ACE2 interface. Sci. Rep..

[72] Khalid Z (2022). In silico mutational analysis of ACE2 to check the susceptibility of lung cancer patients towards COVID-19. Sci. Rep..

[73] Kumar BK (2022). Pharmacophore based virtual screening, molecular docking, molecular dynamics and MM-GBSA approach for identification of prospective SARS-CoV-2 inhibitor from natural product databases. J. Biomol. Struct. Dyn..

[74] Lehrer S, Rheinstein PH (2020). Ivermectin docks to the SARS-CoV-2 spike receptor-binding domain attached to ACE2. In vivo (Athens, Greece).

[75] Eweas AF (2021). Molecular docking reveals ivermectin and remdesivir as potential repurposed drugs against SARS-CoV-2. Front. Microbiol..

[76] Ali SA (2014). A review of methods available to estimate solvent-accessible surface areas of soluble proteins in the folded and unfolded states. Curr. Protein Peptide Sci..

[77] David CC, Jacobs DJ (2014). Principal component analysis: A method for determining the essential dynamics of proteins. Methods Mol. Biol. (Clifton, N.J.).

[78] Wang H (2022). Evaluation of candidatus liberibacter asiaticus efflux pump inhibition by antimicrobial peptides. Molecules (Basel, Switzerland).

