# Supplementary material for: Binding behavior of receptor binding domain of the SARS-CoV-2 virus and ivermectin
Source: Sci Rep. 2024 Feb 2;14:2743. doi: 10.1038/s41598-024-53086-0 (PMC10834942; doi:10.1038/s41598-024-53086-0)
Supplement: Supplementary file 1 — Supplementary Information. [file 41598_2024_53086_MOESM1_ESM.docx]

**Supplementary Materials**


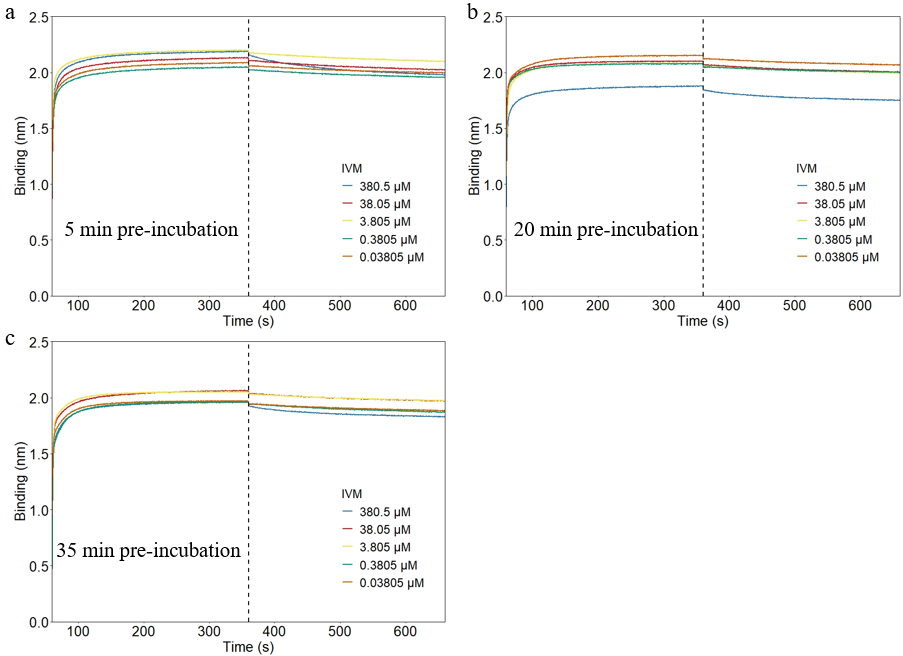
**Supplementary Figure 1.** The binding curves of 5.36 μM RBD and 380.5 μM, 38.05 μM, 3.805 μM, 0.3805 μM, and 0.03805 μM IVM combined with 5 minutes, 20 minutes, and 35 minutes pre-incubation were generated using the basic kinetic analysis. The vertical dotted line indicates the transition from the association step to the dissociation step.

| **ka** |
| --- |
| Various IVM concentration at a single time (5 min): p = 0.0013  Various IVM concentration at a single time (20 min): p = 0.098  Various IVM concentration at a single time (35 min): p = 0.0125  Different time at a single IVM concentration (380.5 μM): p = 0.637  Different time at a single IVM concentration (38.05 μM): p = 0.525  Different time at a single IVM concentration (3.805 μM): p = 0.67  Different time at a single IVM concentration (0.3805 μM): p = 0.144  Different time at a single IVM concentration (0.03805 μM): p = 0.319 |
| **kdis** |
| Various IVM concentration at a single time (5 min): p = 0.008  Various IVM concentration at a single time (20 min): p = 0.0249  Various IVM concentration at a single time (35 min): p = 0.0858  Different time at a single IVM concentration (380.5 μM): p = 0.144  Different time at a single IVM concentration (38.05 μM): p = 0.563  Different time at a single IVM concentration (3.805 μM): p = 0.66  Different time at a single IVM concentration (0.3805 μM): p = 0.699  Different time at a single IVM concentration (0.03805 μM): p = 0.526 |
| **KD** |
| Various IVM concentration at a single time (5 min): p = 0.015  Various IVM concentration at a single time (20 min): p = 0.0025  Various IVM concentration at a single time (35 min): p = 0.0036  Different time at a single IVM concentration (380.5 μM): p = 0.291  Different time at a single IVM concentration (38.05 μM): p = 0.532  Different time at a single IVM concentration (3.805 μM): p = 0.798  Different time at a single IVM concentration (0.3805 μM): p = 0.575  Different time at a single IVM concentration (0.03805 μM): p = 0.425 |

**Supplementary Table 1.** The results from ANOVA testing for each of the pre-incubation time trials. The p values for various IVM concentrations tested at 5, 20, and 35 minutes individually and for 380.5 μM, 38.05 μM, 3.805 μM, 0.3805 μM, and 0.03805 μM IVM concentrations over the span of the three different pre-incubation time periods.


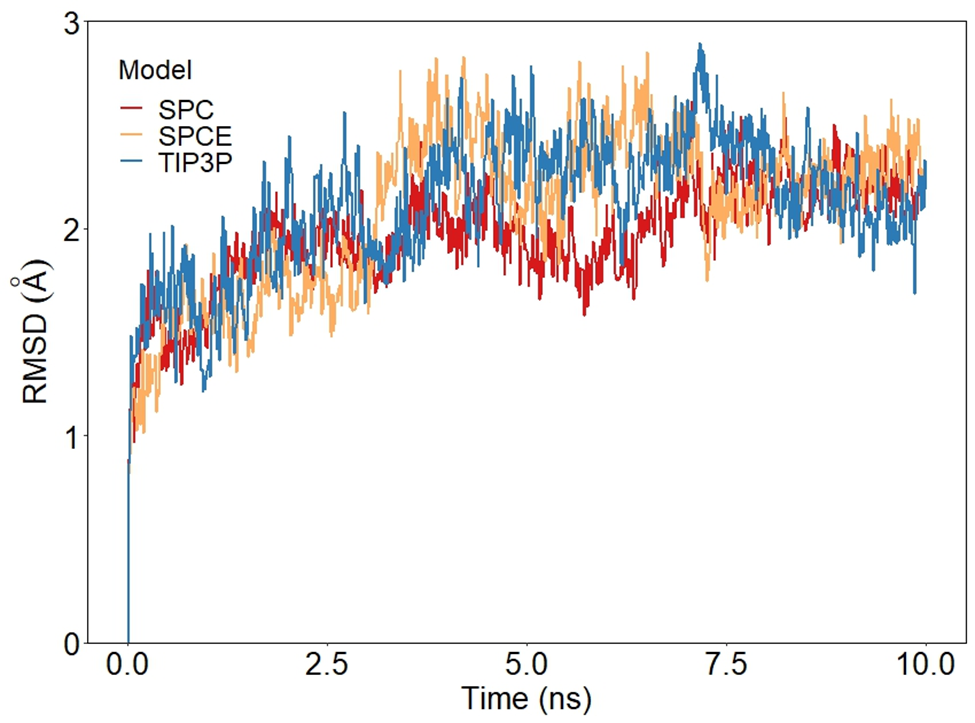
**Supplementary Figure 2.** Protein stability of the RBD with docked ivermectin represented by the Cα RMSD values during 10ns MD simulation with SPC (red), SPCE (yellow), and TIP3P (blue) water models.


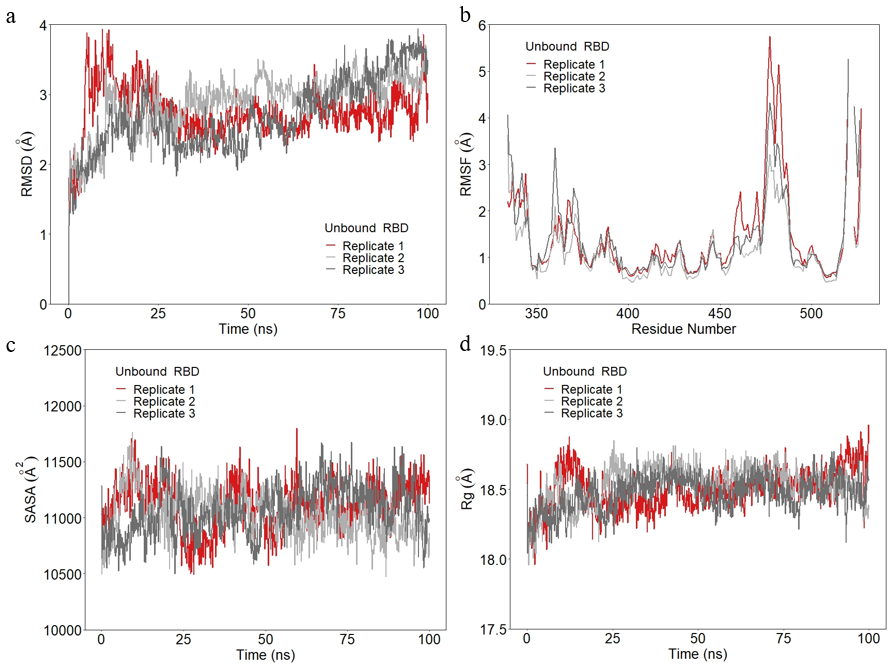


**Supplementary Figure 3.** (a) Cα RMSD values over time for the average displacement change, (b) residue-based protein Cα RMSF over the trajectory for local fluctuation along the protein chain in the unbound RBD, (c) Solvent Accessible Surface Area (SASA) of the unbound RBD protein, and (d) time revolution of radius of gyration (Rg) of the unbound RBD protein during three replicates of 100ns MD simulation. The sets of data that were shown in the main text were marked in red, while the other two replicates were marked in gray for comparison.


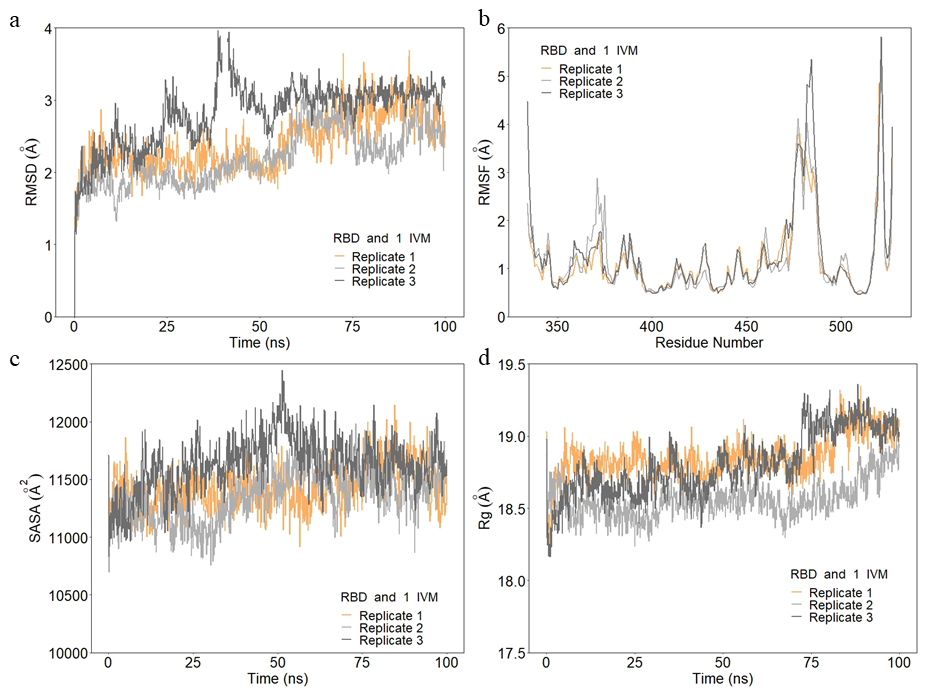


**Supplementary Figure 4.** (a) Cα RMSD values over time for the average displacement change, (b) residue-based protein Cα RMSF over the trajectory for local fluctuation along the protein chain in the RBD protein and one ivermectin, (c) Solvent Accessible Surface Area (SASA) of the RBD protein and one ivermectin, and (d) time revolution of radius of gyration (Rg) of the RBD protein and one ivermectin during three replicates of 100ns MD simulation. The sets of data that were shown in the main text were marked in yellow, while the other two replicates were marked in gray for comparison.


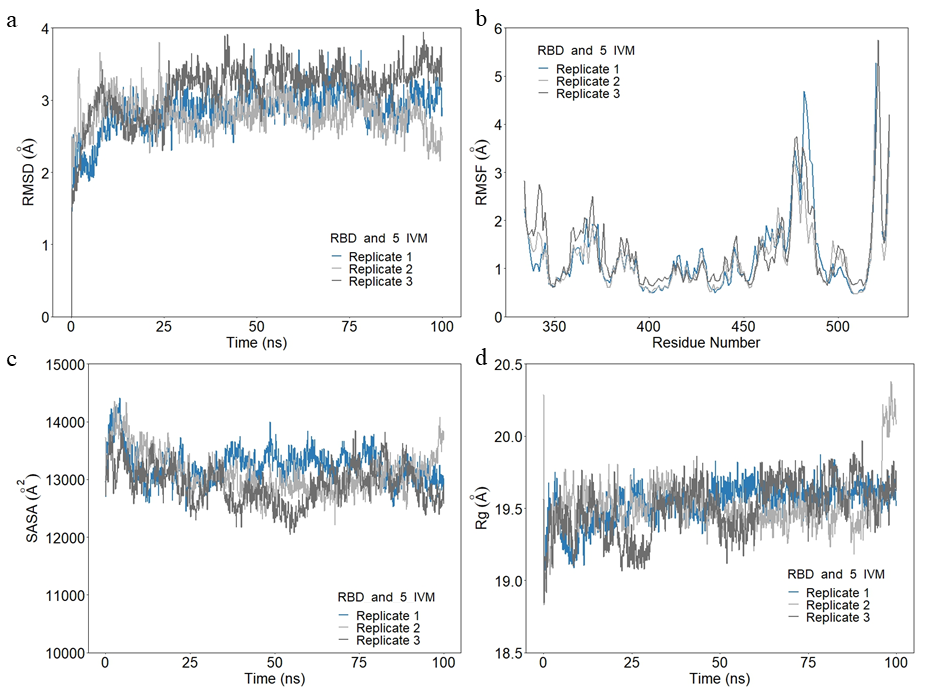


**Supplementary Figure 5.** (a) Cα RMSD values over time for the average displacement change, (b) residue-based protein Cα RMSF over the trajectory for local fluctuation along the protein chain in the RBD protein and five ivermectin molecules, (c) Solvent Accessible Surface Area (SASA) of the RBD protein and five ivermectin molecules, and (d) time revolution of radius of gyration (Rg) of the RBD protein and five ivermectin molecules during three replicates of 100ns MD simulation. The sets of data that were shown in the main text were marked in blue, while the other two replicates were marked in gray for comparison.
